# Mental Hospital Matters

**Published:** 1911-10-21

**Authors:** 


### MENTAL HOSPITAL MATTERS.

(Administrative and Practical Contributions and Photographs invited.)

### Workers in Mental Hospitals: A Call for Justice and Co-operation.

We earnestly desire to invite the co-operation of workers in mental hospitals and asylums everywhere. The position of these workers at the present time, as is clearly proved by the report of the Select Committee on the Asylum Officers' Employment, Pensions and Superannuation Bill, is one which entitles them to the commiseration and support of all workers who are engaged in the service of the voluntary hospitals and kindred institutions. No more striking evidence of the evils, injustices and drawbacks which attach to employment under a State or municipality could be afforded than the facts brought out by the Select Committee. It is cruelly wrong that our mental hospitals, though rate-supported mainly, should at the present time represent for the most part crowded establishments which are sadly understaffed, and where the duties of the staff are especially trying and difficult, having regard to the nature and circumstances of their employment. We have heard much in recent years of sweated nurses, but what language can adequately condemn a system which permits attendants and nurses in asylums to be kept on duty, exclusive of meal-times, for eighty-four hours per week, or twelve hours a day including Sundays? It is quite time that the asylum workers should combine, and that the responsible heads of our mental hospitals, the medical superintendents, should have the support of the Press in bringing about a thorough reform of the existing system, so far as it is defective, with a view to speedily bring to an end the many abuses and evils which at present exist.

Our sympathy is with the medical superintendents and every member of their staffs in regard to the claims which they have for greater consideration and better pay in the majority of instances, with an adequate pension provision and a table of off-duty hours which shall tend to conserve their health and protect them from risks which, as the statistics show in the past, have produced far too many tragedies as represented by ruined mental and bodily health. Let those who are so fond of advocating State or rate-supported hospitals read the report of the Select Committee, and then ask themselves what effect this lamentable neglect in regard to the limitation of hours of duty, the provision of an adequate time for meals under the most comfortable conditions, and the other abuses must have upon the happiness and comfort of the patients. That, however, is another question, and to-day we are concerned with the workers.

We invite correspondence and inquiries from every worker engaged in these institutions. We shall welcome evidence of progress in all departments of a mental hospital, including new methods of classification and treatment, developments in administration, scientific work, and all practical matters, included in departmental organisation. What a helpful, encouraging and interesting series of articles can be written upon the work and management of the mental hospital. Its farm, its system of lighting, of drainage and of water supply! What an infinite variety of practical and instructive facts lie buried in the fruits of the taste, research and artistic efforts of many superintendents in the direction of fittings, furniture, appliances and decoration! The duties which devolve upon the superintendent of a mental hospital are amongst the most responsible, wearing and interesting of all our institutional workers. The responsibility is great; the call for practical knowledge in many directions vast; and the results, as met with in the best asylums and mental hospitals in this country, redound to the credit of the men who devote their lives to this most arduous calling. Another feature of the asylum life is the fire-drill, and a full account, with illustrations, of this branch of the work would prove of great practical interest and value to all institutional workers. There is another side, too, of practical value, and that is the staff of artificers, engineers, carpenters and trained workmen who, under the direction of the medical superintendent, do a great amount of work in these institutions at a minimum of cost.

We have indicated a few of the many sides of asylum life, particulars of which we shall welcome. It is our object to be helpful and instructive as a newspaper, and both ends can be attained if we are fortunate enough to secure the co-operation of all classes of workers throughout the mental hospitals and asylums of the United Kingdom. Their interests are our interests, so let us join hands in united endeavour to remove abuses, to improve, as far as improvement is possible, that which can be changed for the better, and to do justice for the instruction of the public to the many sides of the life and work of our Mental Hospitals. We are convinced that completed public knowledge of these hospitals, what they do and are in fact, would be the shortest means of preventing in future the hardships and discomforts revealed by the report of the Select Committee.

Meanwhile our columns are open to a full discussion of the hardships and claims of the attendants and nurses in Mental Hospitals. We want the facts, but when sent they must rest upon sound evidence or they will not help the cause of the workers. The report of the Select Committee will be made most effective by keeping attention fixed upon its findings and publishing facts in support of the case it contains, and the reasons which may make it but an act of justice for Parliament to go even further than the Select Committee in the legislation which must shortly result. We should like to present the case of the workers in Mental Hospitals as completely as possible.

